# A Novel Protocol for Feasibility and Safety in Early Discharge with ACS

**DOI:** 10.3390/jcm14238373

**Published:** 2025-11-25

**Authors:** Karim Jamhour-Chelh

**Affiliations:** Interventional Cardiology Unit, Cardiology Department, Hospital de la Santa Creu i Sant Pau, 08025 Barcelona, Spain; karim_elkier@hotmail.com

**Keywords:** acute coronary syndrome, early discharge, percutaneous coronary intervention, risk stratification, TIMI, Zwolle index, LATE2ACS protocol, cardiac rehabilitation

## Abstract

**Highlights:**

- Early discharge in low-risk patients with ACS is safe and feasible.
- The LATE2ACS protocol is a novel easy checklist to define low risk for early discharge after ACS.
- A bidirectional management track between CRH and primary care centers is crucial.
- Follow-up after early discharge can be performed telematically without prognostic worsening.
- This new tool will need prospective evaluation to determine its true validation for prognostic value, beyond logistical and functional considerations.

**Abstract:**

Acute coronary syndrome is the primary reason for admission to cardiology services and involves significant resource consumption. Currently, low-risk patients are recommended for discharge within the first 48–72 h, enabling early initiation of cardiac rehabilitation with proper follow-up. This is crucial not only as an efficiency marker but also because current evidence has shown it to be a safe and feasible strategy. Thus, this work aims to provide a comprehensive review of the current evidence on early discharge in acute coronary syndrome and propose a new, intuitive, objective, and easy-to-use tool: the LATE2ACS protocol. This is a narrative, hypothesis-generating review; LATE2ACS is presented as a preliminary, exploratory checklist rather than a definitive or implementable score. Its added value lies in operationalizing post-PCI early discharge decision-making across ACS as a pragmatic bedside checklist that complements, rather than replaces, prognostic scores such as TIMI, GRACE, Zwolle, and CADILLAC.

## 1. Introduction: Background of Early Discharge in ACS

Acute coronary syndrome (ACS) is a leading cause of admission to cardiology services and is therefore resource-intensive. For decades, attempts have been made to define risk factors correlated with adverse cardiovascular events (MACE) in patients with ACS. Since Califf et al. [1] presented the Angina Score in the 1980s, many scoring systems and calculators have been proposed to predict MACE. Current recommendations support using the TIMI score [2,3] (Table 1) and the Global Registry of Acute Coronary Events (GRACE) [4]. However, it is important to note that the GRACE score is based on patients who were primarily treated with fibrinolysis in the pre-percutaneous coronary intervention (PCI) era.

Since PCI has become the gold standard in the treatment of ACS, other scoring systems have emerged. However, their clinical use remains limited, possibly due to more complicated assessment, the need for special calculators, and greater diversification based on ACS subtypes. Predicting patient outcomes provides practitioners with a tool to adjust their therapeutic approach. The systematic implementation of the regional STEMI network and easy access to angioplasty in less than 2 h have led to a significant reduction in morbidity and mortality [5,6]. Therefore, hospital stays for ACS with ST-segment elevation (STEMI) have traditionally been necessary to monitor arrhythmic and mechanical complications during infarction or after revascularization. However, the extension of primary angioplasty as a standard treatment and the creation of rapid care networks have significantly improved survival rates and decreased complications associated with these events [7,8].

These factors, particularly in light of the COVID-19 pandemic, have prompted a reassessment of the necessity for a shorter hospital stay that can provide a safer balance between effective patient management for ACS and efficient use of healthcare resources.

The aim of this study was to present a narrative review of early discharge in ACS and to propose a preliminary, exploratory checklist (LATE2ACS). To that end, we will be reviewing the risk scores published in previous studies and proposing a new risk index to establish early discharge in patients with ACS. This index will attempt to include those with STEMI as well as those with non-ST-segment elevation (NSTEMI). Thus, the main objective is to present a new, versatile tool that reflects the published evidence and facilitates the early discharge of patients undergoing percutaneous coronary intervention (PCI) in the context of ACS. In keeping with its narrative scope, this manuscript does not purport to deliver a systematic review or immediate implementation guidance, but to organize contemporary evidence and propose an exploratory framework to be prospectively tested. Within this context, LATE2ACS contributes by integrating angiographic success, clinical stability, simple imaging and laboratory thresholds, and follow-up feasibility into a single pragmatic checklist distinct from TIMI/GRACE (presentation risk) and Zwolle/CADILLAC (post-PCI prognosis).

## 2. Risk Scores to Establish Early Discharge in ACS

The 2019 European Society of Cardiology (ESC) Guidelines recommend evaluating hospital discharge within the first 48–72 h for low-risk patients who can begin cardiac rehabilitation (CRH) early with proper follow-up [9]. Studies have even evaluated the safety of discharge strategies in less than 48 h (very early discharge) [10,11,12,13,14]. Patients at low risk of major adverse cardiac events (MACE) may benefit from earlier CRH and shorter hospitalization without increasing the risk of death or recurrent acute coronary syndrome (ACS). Thus, approximately one third of patients with ACS have a low-risk profile, which allows for a relatively safe discharge home before 72 h. This is important not only as an efficiency marker but also because patients report greater satisfaction [11,15,16,17].

In European countries, only 28% of patients who were eligible for early discharge actually discharged early, compared to the United States (USA) where the early discharge rate for patients with ACS is 60% [18,19]. Hospitalization for ACS in Europe is significantly longer, with only 16% of patients receiving early discharge outside the USA. However, the readmission rate within 30 days is significantly higher in the USA. Additionally, there is a higher prevalence of comorbidities such as advanced age, diabetes, hypertension, atrial fibrillation, and multivessel coronary artery disease (CAD) [20].

The Zwolle index was developed and validated as a prognostic score to identify patients with ST-elevation myocardial infarction (STEMI) who are at low risk of complications and can be safely discharged within 72 h [21]. This index provides a simple method (Table 2) to select low-risk patients early during hospitalization based on age, success of percutaneous coronary intervention (PCI), and the presence of signs of heart failure (HF) [12,14,22,23]. The ESC clinical practice guidelines recommend using the Zwolle index to select patients eligible for early discharge in the setting of STEMI.

This would also enable earlier access to CRH programs with structured and systematic follow-up [9]. Recent ESC recommendations for the management of ACS establish guidelines for monitoring uncomplicated ACS with 24 h post-PCI follow-up, which could be extended beyond 24 h in case of complications [9].

**Figure 1:**
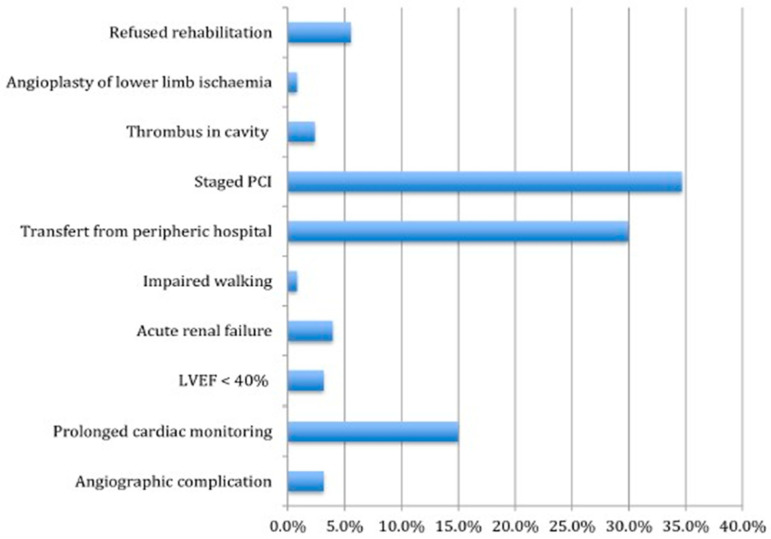
Main causes preventing early discharge in patients with ACS. Adapted from the 2016 publication by Laurence M.E. et al. [24] in which the 2 most important reasons preventing early discharge were: (a) requirement for PCI in the second stage and, (b) transfer from another external hospital. Therefore, it is very important to consider all these factors that are not related to the patient’s clinical profile and can hinder early discharge management if the patient’s risk index is considered. Legend: ED = early discharge; PCI = percutaneous coronary intervention; LMCA = left main coronary artery.

It is important to note that a significant portion of patients who meet the criteria for early discharge according to the Zwolle index end up having to extend their hospital stay. This is often due to the need for revascularization therapy in the second stage or additional tests and adjustments to medical treatment. The main barriers that often prevent early discharge after PCI are summarized here (Figure 1) [24]. There has been controversy for years regarding whether the lesion should be completely revascularized during the index procedure, particularly in the context of cardiogenic shock [25,26]. However, recent studies support complete revascularization over treating only the culprit lesion [27,28].

Several scoring systems have been proposed to predict the outcomes of patients with ACS. In some cases, these scores may help to reduce hospital stays without compromising patient prognosis. However, the use of these scoring systems in clinical practice is limited. A recent study conducted by a group from Prague [29] evaluated and proposed a series of predictors to identify patients with ACS who have a low-risk profile (Table 3). The study analyzed a cohort of 1420 patients diagnosed with ACS from 2018 to 2020. A univariate analysis was performed on patients treated by PCI, comparing each predictor with the 30-day mortality rate. Out of all the variables analyzed, 11 correlated significantly with 30-day survival (Table 3). This study highlights the importance of combining clinical, echocardiographic, and angiographic parameters to create a low-risk prognostic profile. This approach can help identify patients with ACS who are suitable for early and safe discharge.

The Zwolle index defined the low-risk profile with only six variables (Table 2) from a cohort of 1791 patients recruited from 1994 to 2001 [21]. Although the results were promising for selecting patients for early discharge, severe contraindications were found in almost 17% of patients that led to prolongation of hospital stay: HF, malignant arrhythmia or AV block, pericardial effusion, cardiac surgery, intra-aortic balloon, fever, acute thrombosis, and renal failure [21,29].

In 2005, another risk score called CADILLAC [30] was proposed that uses seven variables weighted proportionally according to odds ratios: age, Killip class, baseline LVEF, anemia, renal failure, 3-vessel CAD, and final TIMI flow after PCI (Table 4). Thus, it was the first study to propose the importance of LVEF as the single most powerful predictor of survival after ACS. In the study that proposed this scoring system, more than half (56.5%) of the patients were identified as low-risk, with mortality rates of 0.1% at 30 days and 0.8% at 1 year. However, the limitation of this study is the presence of somewhat strict exclusion criteria such as symptoms of 12 h of evolution, cardiogenic shock, failed thrombolytic treatment, need for multivessel PCI, bleeding diathesis, and severe comorbidities with a life expectancy <1 year [30].

The most recent risk score to date, published in 2012, the ACUITY-PCI (Table 5), consists of a complex scoring system evaluating clinical, laboratory, electrocardiographic, and angiographic parameters in patients admitted for NSTEMI [31]. In a novel way and unlike previous ones, this score took into account the complexity and characteristics of the angiographic lesions, as well as creatinine clearance or troponin elevation. Again, it exemplifies the need for a good combination of multidimensional data to obtain a real profile that constitutes the low risk of our patients. The caveat of the ACUITY-PCI is that, unlike the CADILLAC score (Table 3), it differentiates between NSTEMI and STEMI. Therefore, it would be interesting to establish a risk scale that encompasses ACS as a whole, seeking its applicability in a more heterogeneous population without such strict and restrictive inclusion criteria as described in the previous paragraphs. Thus, it seems that the criteria shown by the team of D. Bauer et al. [29] show a more pragmatic and current perspective with promising results when compared with the risk scales designed for the population with ACS treated by PCI.

Along these lines, one of the first prospective studies dealing with early discharge was recently published. Using the low-risk profile criteria proposed by the ESC (Table 6), a total of 600 patients were prospectively included between 2020 and 2021. Follow-up at discharge, carried out by a multidisciplinary team, included a telephone call 48 h after discharge, as well as virtual reviews at 2, 6, and 8 weeks and finally at 3 months. All of this was achieved through a smartphone app with the option of additional virtual assessments if necessary. The study sample was compared with a historical cohort of 700 patients with similar characteristics who, between 2018 and 2021, had been discharged in >48 h. The study results were favorable, achieving a median hospital stay in the early discharge cohort of 24.6 h. There was no loss to follow-up (median 271 days), with only two deaths (which were from COVID-19), and an overall MACE incidence of 1.2%, with no statistically significant differences between the two groups [11].

Thus, following the criteria for low-risk STEMI established by the ESC (Table 6), these patients would be discharged within 24 h. Even so, as stated in these guidelines, this means that there is less time to educate our patients, adjust optimal medical treatment, and, in short, adequately establish the basis for successful secondary prevention. Proper patient education in their pathology will be the key to a good subsequent follow-up and adequate therapeutic adherence. For this reason, it is crucial [11] to offer a structured follow-up program in CRH. Therefore, everything indicates that the low MACE rate observed in the very early discharge group is closely related to adequate patient selection and a systematic follow-up plan associated with a structured and robust CRH program [9,11].

Likewise, in several studies carried out in Spanish centers, there has been relatively good experience with protocols (Table 7) for early discharge [32]. Thus, several studies have been carried out, including the FASTEST score [33] to establish early discharge between 24 and 48 h designed by colleagues at the Gregorio Marañón Hospital in Madrid, which was later validated by the Santiago de Compostela group [34]. This score considered radial access, Killip I status, TIMI 3 final flow, LVEF > 50%, and age < 65 years, with creatinine < 1.5 mg/dL and absence of LMCA disease. In some ways these are remarkably similar to the criteria established by the Zwolle index with the exception that they raise the cut-off point for serum creatinine and LVEF. In any case, it seems that estimating the glomerular filtration rate provides relevant information for the acute phase prognosis of patients admitted for ACS, so this may need to be added in the evaluation of these patients [29,35].

## 3. LATE2ACS Protocol to Achieve Early Discharge in ACS

Based on the current evidence regarding early discharge in patients with STEMI and NSTEMI, this comprehensive narrative review has selected the following parameters. An intuitive index or algorithm has been established to identify patients with ACS with a low-risk profile. Thus, a tool has been developed using parameters validated in previous studies to facilitate the task of establishing early discharge in patients with ACS. This tool is applicable to both STEMI and NSTEMI; in NSTEMI, eligibility is conditional and individualized due to diagnostic timelines and staged strategies.

NSTEMI scope (operational rule). Early discharge eligibility in NSTEMI is conditional and requires: (I) PCI performed within <24–36 h of diagnosis; (II) no staged revascularization planned (no deferred PCI or scheduled CABG); (III) no PCI complications (e.g., sustained no-reflow, perforation, acute stent thrombosis, type 4a MI) and no LMCA culprit; (IV) early echocardiography ≤ 24 h with LVEF ≥ 45% and absence of anterior wall akinesia; (V) eGFR > 60 mL/min/1.73 m^2^, Hb ≥ 11 g/dL, glucose < 270 mg/dL; and (VI) final TIMI 3 flow in the treated vessel. If any of these conditions is not met, early discharge is not recommended.

The LATE2ACS protocol (**central illustration**) arises as a synthesis and response to all the ideas that were set out previously and that possibly in the ESC guidelines are not very concise despite the evidence currently available. Thus, this LATE2ACS protocol is designed to assess those patients admitted for ACS who present a low-risk profile for MACE and who can be discharged home within 48–72 h in a safe way, thus favoring earlier access to the CRH program, better management of resources, and greater patient satisfaction. Thus, we present a series of criteria that would make up this low-risk profile in patients with ACS.

As can be seen in the **central illustration**, an index or protocol is proposed that basically consists of eight criteria that are summarized in a simple and intuitive acronym that will facilitate their application by the cardiologist to propose early discharge in the context of ACS. The seven letters of LATE2ACS group eight pragmatic criteria for bedside use, forming the English phrase “LATE2ACS” which stands for “late to acute coronary syndrome”, and it consists of the following **(central illustration)**. Importantly, LATE2ACS is a bedside checklist—not a points-based prognostic score—with simple decision logic: all mandatory items met and no planned staged revascularization imply eligibility for an early discharge pathway in appropriately selected settings.

- **Lungs**, which is represented by the letter “L” for lungs. It represents the presence of pulmonary congestion which, in short, defines the congestive state of the patient with ACS and is evaluated using the Killip-Kimball scale (KK) proposed in the 1980s and which has shown its prognostic value in patients with ACS in whom early discharge is considered. Thus, in order to consider early discharge, our patient should be in a stable situation in the absence of HF: Killip-Kimball class I.
- **Age and autonomy,** which is represented by the first “A” in the acronym. It refers to the age of the patient and baseline status. Age is a clear CVRF and studies have shown that it is safe to propose early discharge at ages below 75–80 years. Although the first scores proposed more restrictive ages, with the aging of the population and better access to primary PCI, the overall prognosis of these patients has improved. On the other hand, another relevant factor that defines this criterion is the baseline situation, “autonomy”; early access to CRH should be guaranteed, as well as care and maintenance of optimal medical treatment during follow-up. Therefore, all patients who are considered for early discharge should have acceptable independence for activities of daily living without significant signs of frailty and acceptable social and/or family support.
- **TIMI final flow**, represented by the letter “T” of TIMI flow, which defines the final flow state after PCI. From the pathophysiological point of view, it is essential to obtain TIMI 3 final flow, which indicates complete restoration of flow in the culprit coronary artery and, in short, acute treatment of this ACS, limiting the ischemia time.
- Ejection fraction, represented by the letter “E”, with LVEF being the main prognostic factor in patients with ACS during follow-up. This is so much the case that those patients with LVEF ≤ 35% present a significantly higher risk of debuting with sudden cardiac death in the first month of follow-up after the index event. Thus, it is essential that all patients considered for early discharge have at least LVEF > 45% estimated by transthoracic echocardiography [11,29,30,32].
- **2A** comes to represent the second letter “A” which has a double meaning (2A), “the 2 absences”:
- **Absence of anterior wall akinesia**, thus ruling out those patients with indirect data of extensive AMI, prolonged ischemia with possible adverse remodeling, which will probably be accompanied by a significant decrease in LVEF and therefore should be monitored and cared for longer due to the inherent risk of complications. Thus, a patient with akinesia in the theoretical territory of the left anterior descending (LAD) artery cannot be admitted for early discharge [21,24]. This item reflects its historical origin in the Zwolle index (anterior AMI); recognizing inter-observer variability across imaging metrics, contemporary practice may reasonably rely on standardized early TTE with LVEF (e.g., ≥45%) and/or GLS as pragmatic surrogates where available.
- **Absence of laboratory abnormalities**, which is basically based on the indirect detection of comorbidities that may presage greater risk secondary to ACS therapies or that go beyond the coronary event itself. Of the parameters and comorbidities considered, only three have been shown to be related to the low-risk profile: hemoglobin, glycemia, and renal filtration rate. Paradoxically, hemorrhagic events have not been shown to be associated with early discharge protocols. Thus, according to the literature [11,24,29,35], early discharge may be considered in those patients who do NOT present anemia with Hb < 11 g/dL, glycemia > 270 mg/dL (>15 mmol/L), and eGFR < 60 mL/min/1.73 m^2^ [2]. Another important aspect from a laboratory perspective is that the patient has reached peak troponin, which is a marker of myocardial damage. In the setting of STEMI, those who died at 30 days and at 1 year had significantly higher peak troponin levels than those who survived. Peak troponin is also inversely proportional to LVEF, with higher troponin levels associated with lower LVEF [35]. In the context of ACS, blood troponin levels are elevated with a time lag. Therefore, it is highly likely that ongoing ischemia has largely stopped after coronary flow has been restored after PCI, when peak troponin has been reached at the moment when discharge is considered. Studies published previously have not identified a troponin level cut-off value to indicate higher or lower risk in early discharge. However, elevated blood troponin is an adverse prognostic parameter itself. In stable patients with STEMI who have undergone successful primary PCI, the long-term LVEF and infarct size are closely correlated with both serial and peak concentrations of high-sensitivity cardiac troponin T [36]. Operationally, documenting a reached peak followed by an early down-trend (e.g., one value 6–12 h post-PCI, or two values 3–6 h apart) supports that acute injury is not ongoing and can aid next-day discharge decisions in otherwise suitable cases. “Peak reached” is defined by one sample 6–12 h post-PCI showing an absolute decrease from the prior value or two samples 3–6 h apart with a downward trend. No fixed cut-off is used; the criterion captures post-reperfusion decline as a signal of stabilized myocardial injury.
- **Coronary complications**, represented by the “C” of “ACS”, which makes it very intuitive. This item defines a situation of successful PCI (which was already defined by the TIMI 3, described as a “double check”) but also rules out the presence of complications derived from the procedure such as slow/no-reflow phenomena, coronary dissection requiring more stent placements, or a conservative approach and subsequent re-evaluation. There should be complete revascularization, and it can be considered in the case of incomplete revascularization as long as the remaining lesions are not significant (stenosis < 70%) and revascularization is not pending a second time in an imminent fashion. Patients with culprit lesions in the left main coronary artery (LMCA) should be ruled out for early discharge. Arrhythmias derived from the procedure are not considered a complication. Planned staged PCI or CABG in the near term excludes early discharge; non-culprit residual disease can be acceptable when it is angiographically non-significant (e.g., <70%) and clinically non-ischemic. Periprocedural myocardial infarction (type 4a), frequently related to distal embolization or microvascular obstruction, carries adverse short- and long-term prognostic implications even after technically successful PCI and should exclude candidates for early discharge [37].
- **Shock and support**, represented by the “S”, referring to the shock situation encompassing all those situations in which the patient is in clinical and/or hemodynamic instability (HDI) requiring medical and/or mechanical support by cardiopulmonary resuscitation (CPR) maneuvers due to cardiopulmonary arrest (CPA), inotropic treatment, vasoactivity, appearance of ventricular arrhythmias > 24 h of PCI, or persistent arrhythmias, and ventilatory and/or mechanical circulatory support. In short, it is, as a whole, again a “double check” for all those processes that go beyond the definition of KK-IV.

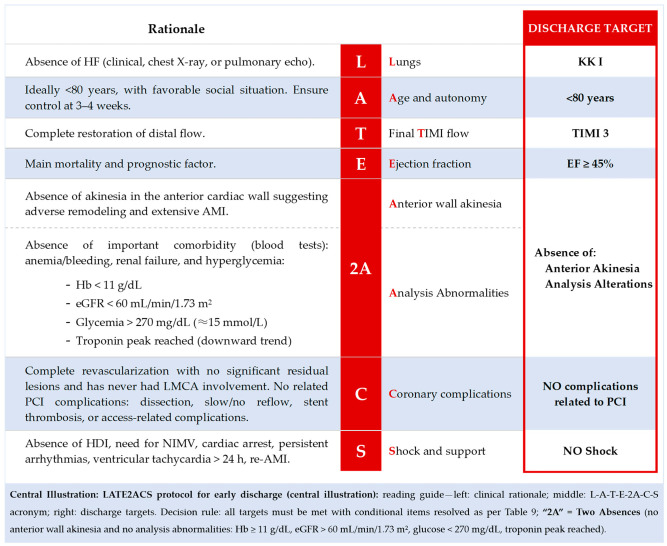


In summary, according to this new protocol **(central illustration)**, early discharge can be considered within 48 h in those patients who have had ACS regardless of its location and/or the number of affected vessels as long as there is no LMCA culprit lesion (Table 3) [29]. In addition, there should be an uncomplicated PCI with TIMI 3 flow and complete revascularization, and in the case of residual lesions, they should be non-significant (stenosis < 70%). Likewise, the presence of LV dysfunction (LVEF < 45%) and/or the presence of akinesia in the LAD artery territory should be ruled out by TTE. Early discharge will be considered in a patient without significant comorbidities whose admission is without mechanical and/or arrhythmic complications and without requiring support of any kind.

Thus, the LATE2ACS protocol presents relevant aspects for establishing the low-risk profile after ACS in an intuitive and concise manner. Although these parameters have been supported by previous studies, they have not been assessed together in a prospective study. This fact limits the prognostic value of this new tool to some extent. Therefore, while this tool summarizes previous published evidence, it should be evaluated in prospective studies to determine the weight of each variable. It is unclear whether meeting all eight criteria in the LATE2ACS protocol is mandatory for early discharge or if there is a cut-off point.

### 3.1. LATE2ACS: How Was It Made?

In line with a narrative, hypothesis-generating approach, items were selected from consistently reported low-risk features across guideline documents and key cohorts and then mapped into a bedside checklist; no formal weighting, derivation, or calibration was performed.

The aim of this review was to design a protocol that would be useful across the spectrum of ACS: STEMI, NSTEMI, and unstable angina (UA). Most risk scores have been designed and validated in the context of STEMI, with the exception of the TIMI risk score [2,3], in which there is a targeted score to assess risk in patients with UA/NSTEMI differentiated from patients with STEMI (Table 1). A similar situation occurs with the ACUITY-PCI risk score, which was developed specifically for patients with NSTEMI [31]. The GRACE 2.0 risk score [4] was developed for ACS risk stratification and not so much to assess the safety of early discharge in patients undergoing PCI. Furthermore, GRACE 2.0, as with other risk scores such as ZWOLLE, was developed and validated in the pre-PCI era, so the current context is not actually the same.

The vast majority of risk calculators and recommendations have been published in the context of STEMI [21,25,30,32], leaving those patients admitted for NSTEMI/UA without clear guidance. The current recommendations of the ESC and the consensus document advocate a very general set of recommendations (Table 6) based on a study published in 2022 by Bauer D, et al. (Table 3) [29]. Considering all the available evidence on early discharge in patients with ACS, including those with STEMI and NSTEMI/UA, this review aimed to create a mnemonic tool that simplifies the validated data from previous studies.

The goal was to create a clinical tool that could determine a low-risk profile in the context of ACS within the first 24 h of admission, allowing for early discharge within 48–72 h. The variables used would be clinical, laboratory analysis-based, and echocardiographic in nature. According to ESC guidelines, routine echocardiography is recommended [26]. Additionally, in all patients with ACS, troponin serology is performed, along with a complete screening for cardiovascular risk factors [26]. Therefore, this new tool should be integrated into routine clinical practice without overburdening the responsible physician.

Based on evidence collected up to 2025 (Table 8), previous studies have shown that age, left ventricular ejection fraction, presence of multivessel coronary artery disease, anemia, hyperglycemia, renal dysfunction, and heart failure status in the context of ACS are the parameters with the most significant prognostic implications for early discharge of patients with ACS. A color-coded comparative matrix illustrates the overlap across prior tools; repeatedly validated core components were retained in LATE2ACS, whereas context-dependent items were kept optional to preserve usability. Thus, it is evident that the new LATE2ACS protocol reflects the evidence of eight risk scores or inclusion criteria (Table 8) that define the low-risk profile and, therefore, suitability for early discharge in the context of STEMI and NSTEMI. Although this new tool has not been prospectively evaluated with each of the variables it includes, it appears to be an appropriate and simple tool for comprehensively and intuitively assessing patients suitable for early discharge.

It is interesting to note the particular complexity of the ACUITY score [31]. As previously described, it is a complex score defined by findings related mainly to angiographic parameters, taking into account the presence of diffuse CAD, lesion length, and the presence of bifurcation lesions, as well as taking into account patients with renal insufficiency or diabetic patients treated with insulin. Given its complexity, it can be seen (Table 8) that the concordance within this risk score is significantly lower compared to the others for the new LATE2ACS protocol.

Therefore, the LATE2ACS protocol was developed to simplify the evidence and provide a safe and easy-to-remember tool for early discharge of ACS patients. This protocol serves as a mnemonic rule summarizing the safety evidence for early discharge in the context of ACS.

### 3.2. Operationalization of LATE2ACS

LATE2ACS is applied at the bedside as a step-wise pathway from the index PCI to discharge, combining mandatory clinical/procedural gates—Killip–Kimball I, final TIMI 3 flow, no PCI complications, no planned staged revascularization, LVEF ≥ 45%, and eGFR > 60 mL/min/1.73 m^2^—with a conditional domain (“2A”) that requires no anterior wall akinesia and laboratory stability (Hb ≥ 11 g/dL, glucose < 270 mg/dL) with troponin peak reached (declining trend). An organizational prerequisite (nurse call at 48–72 h, virtual reviews at week-2 and week-6, cardiac rehabilitation ≤ 14 days, 24/7 ED fallback) must be secured before discharge.

**Figure 2:**
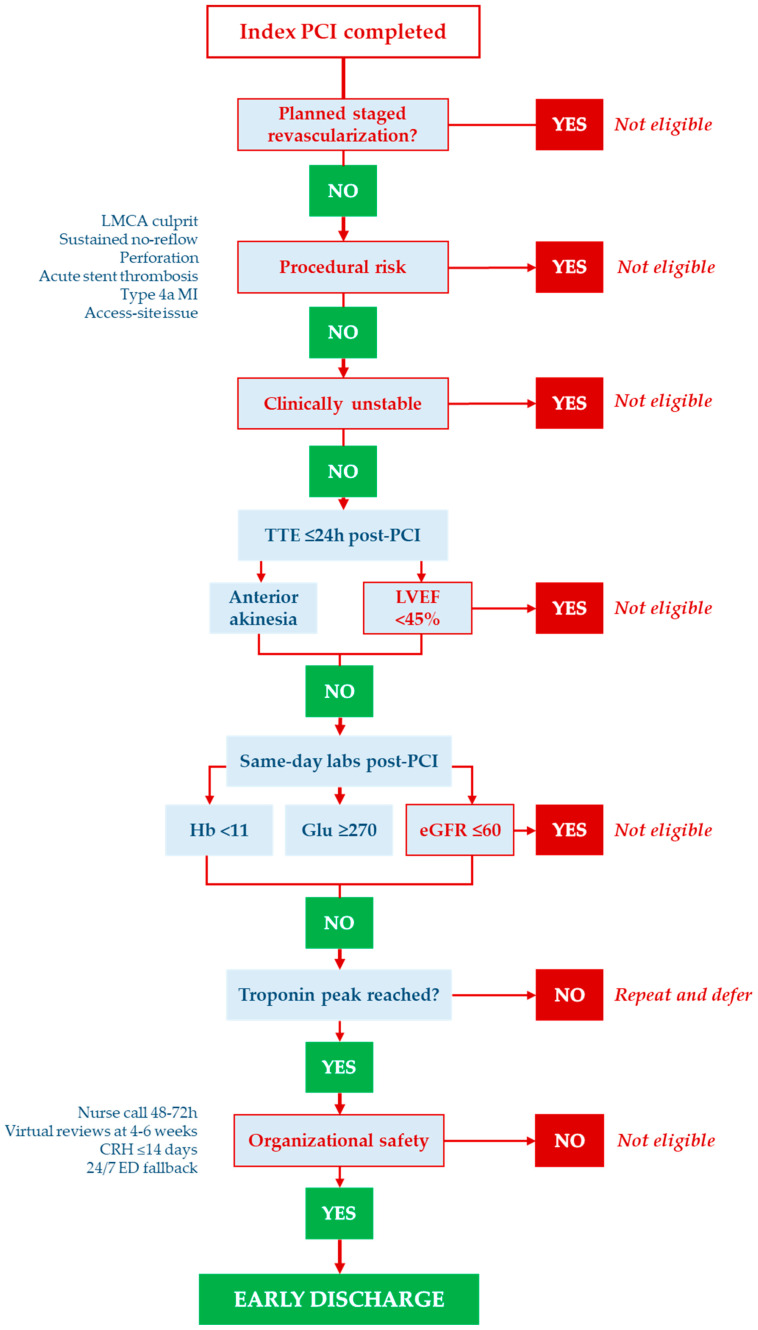
**Step-by-step operational flow for LATE2ACS from index PCI to early discharge.** Mandatory items (red ones) must be met; conditional items (C) must be resolved or have a clear corrective plan before discharge (blue ones); organizational mandatory (M-org) denotes the required safety net. The flowchart operationalizes the **central illustration** and pairs with the LATE2ACS decision table (mandatory vs conditional). Key: M = mandatory; C = conditional; M-org = organizational mandatory.

The operational flowchart (Figure 2) details the decision nodes and timing (target discharge < 48–72 h), while the decision table (Table 9) maps each item as mandatory vs conditional; exclusion criteria (Table 10) are enforced at the staged-revascularization, procedural-risk, and organizational gates.

### 3.3. Safety and Feasibility in Early Discharge

One of the consequences of the improvement in the management of STEMI has been the reduction in hospital stay length. The benefits of a shorter hospital stay are evident and include, among others, greater patient satisfaction, a reduction in complications derived from prolonged hospitalization, and, definitively, a reduction in healthcare costs. All this is of paramount importance in the wake of the consequences of the COVID-19 pandemic. As early as 1998, the PAMI-II study [38] demonstrated the safety and cost-effectiveness of early discharge 72 h after STEMI in low-risk patients. In 2015, a randomized study was published with 100 patients in which the safety of the strategy of early discharge at 48–72 h was demonstrated in those patients considered low-risk according to the Zwolle index [22]. These studies and many others are reflected in the clinical practice guidelines of the ESC, with a level IIa A recommendation for discharge at 48–72 h in low-risk patients [9]. For operational clarity, Table 10 details the exclusion criteria that gate the LATE2ACS pathway (e.g., LMCA culprit, staged revascularization, significant PCI complications, hemodynamic/respiratory support, persistent arrhythmias, or the absence of a secured follow-up pathway).

Discharge home of low-risk patients after primary PCI for STEMI at 48–72 h is a safe and feasible strategy based on meta-analyses and large observational studies [9,10,12], so much so that the aforementioned study by Rathod et al. [11] suggests that it is possible to be even more aggressive by implementing very early discharge before 48 h with a reduction in hospital stay length, thus increasing the availability of beds for hospitalization of new patients and savings in healthcare costs. This is in agreement with previous studies in which discharge within 24–48 h after STEMI has been proposed without increasing MACE rates [13,14,39,40]. Similarly, and with increasing importance, ESC clinical practice guidelines insist on individual risk assessment based on comorbidities, functional status, and social support [9]. Therefore, in this combination of parameters shown by previous risk scales, this sphere of pathology that transcends cardiac risk alone should also be taken into account, and the patient’s clinical situation should be comprehensively encompassed, taking into account their family and socioeconomic and geographic situation to determine the true low-risk profile. This is important because it is assumed that close follow-up will be more complex in those patients who live far from a hospital and therefore telemedicine and telematic follow-up can be crucial tools for maintaining the safety of early discharge of these patients.

What is striking about this study by Rathod KS, et al. [11] is the absence of inclusion of criteria considered in previous scores to define low-risk patients. Thus, ischemia time was not taken into account, although the median symptom-to-balloon time was remarkably acceptable at about 80 min. On the other hand, patients with anterior AMI and/or LVEF 40–50% were not excluded, when they were previously taken into consideration in several of the aforementioned risk scores [26,29,30,32]. Furthermore, perhaps the crucial aspect in relation to the management of these patients is the completely virtual post-discharge follow-up by telephone calls and the use of a smartphone app, which made it possible to adequately establish all secondary prevention management, saving visits to the hospital.

## 4. Discussion and Limitations

This study reviews the published evidence on early discharge in patients admitted for ACS. Most prior work has centered on STEMI, often excluding NSTEMI owing to the lack of rapid-access pathways and the inherent diagnostic delays not anchored to clear electrocardiographic criteria. Nevertheless, several investigations and risk indices have evaluated both phenotypes, and their comparative signals are discussed herein.

A major contribution of this review is not only to synthesize prior studies but also to advance a pragmatic bedside index—LATE2ACS—intended to support clinicians when considering early discharge. For STEMI, contemporary evidence suggests that discharge within 24–48 (up to 72) hours can be safe in carefully selected, low-risk patients after primary PCI, with very low short-term event rates and potential efficiency gains. Observational cohorts and structured-pathway implementations consistently show favorable outcomes for ≤48–72 h discharge when uncomplicated PCI yields final TIMI 3 flow and no periprocedural complications [41]. In addition, risk-tool–guided selection (e.g., Zwolle/CADILLAC frameworks) continues to demonstrate acceptable discrimination for identifying candidates suitable for shorter length of stay in the primary PCI era [42].

By contrast, NSTEMI pathways face practical barriers that complicate implementation within 24–48 h. Time from admission to invasive evaluation is variable—even under preferential scheduling—and in-hospital complications accrue beyond the index day, which historically translates into longer median length of stay and a higher proportion of early post-admission events compared with STEMI fast-tracks [43]. In this context, the present framework explicitly treats NSTEMI eligibility as conditional and individualized, acknowledging that staged revascularization, delayed imaging, and comorbidity optimization often preclude very early discharge.

Beyond mortality or MACE, safety endpoints should capture common, patient-relevant complications after PCI and early discharge. The BARC standardized definition enables consistent bleeding ascertainment and comparability across studies; centers should predefine BARC ≥ 2 as a core safety outcome and consider ARC-HBR features when interpreting bleeding risk in borderline cases [44,45]. Likewise, acute kidney injury should be monitored using KDIGO criteria (creatinine and urine output), given periprocedural nephropathy and hemodynamic shifts in the first 48–72 h; a small creatinine rise may still qualify as AKI Stage 1 per KDIGO and should inform discharge timing and early laboratory follow-up. These standardized frameworks (BARC/KDIGO) also facilitate cross-study synthesis and prospective validation plans [46].

Implementation is system-dependent. Structured early-discharge pathways typically bundle patient education, medication reconciliation, and secured follow-up (tele-contact at 48–72 h plus early clinic or virtual visits), often alongside rapid referral to cardiac rehabilitation. Experience from service-level roll-outs indicates that the combination of short length of stay and prompt rehabilitation access is feasible and aligned with guideline-oriented care when delivered within a hub-and-spoke PCI model [47]. Moreover, emerging randomized and prospective studies suggest that telemedicine-enhanced follow-up after ACS can improve patient experience, risk-factor control, and potentially reduce unplanned utilization—an infrastructure particularly relevant when discharge occurs earlier in the index hospitalization [48]. These organizational prerequisites (reliable tele-channels, proximity to PCI-capable centers, and rehabilitation capacity) are unevenly distributed and should guide center-level adoption and equity considerations.

Limitations deserve emphasis. As a narrative, hypothesis-generating synthesis, this review is prone to selection and confounding bias. Observational cohorts supporting very early discharge often rely on low-risk case-mix and center expertise, which may limit generalizability. Event rates in modern PCI series are low, and even small absolute risk differences can have practical relevance when scaled to population level. While prior scores (e.g., Zwolle, CADILLAC) support risk-guided shortening of stay, their positive findings are not directly transferable to a new checklist without prospective evaluation [42]. Accordingly, prospective validation of LATE2ACS is essential and should include: (a) prespecified BARC and KDIGO endpoints; (b) 30-day unplanned readmissions and ED visits; (c) medication discrepancies at first follow-up; and (d) patient-reported outcomes capturing symptoms and self-care. Tele-monitoring readiness and rehabilitation access should be treated as organizational co-interventions, not mere background features, because they likely mediate both safety and patient experience [47,48].

Finally, future work may compare same-day/≤24 h discharge vs 24–48 h and 48–72 h strategies in STEMI low-risk cohorts, where pilot data already hint at safety and system benefits when stringent selection and follow-up are ensured; stepped-wedge or cluster designs could pragmatically test scalability across networks with heterogeneous resources [42]. In NSTEMI, hybrid pathways prioritizing expedited angiography, early echocardiography, and pre-scheduled tele-visits may help narrow timelines and identify the subset suitable for early discharge without compromising safety [43].

Given the nature of this study, the tool has not been specifically evaluated prospectively, so its implications in risk stratification for patients with ACS are limited. The positive results obtained in previous risk scores cannot be extrapolated to this new risk index. However, the LATE2ACS index is based on clinical parameters that have shown adequate risk stratification in establishing low-risk profiles in patients with acute coronary syndrome. Therefore, it is essential to confirm the validity of this new tool in prospective studies and ensure its practical usefulness in daily clinical practice. Any prospective evaluation should predefine safety endpoints beyond MACE (e.g., bleeding, acute kidney injury, unplanned readmissions, medication discrepancies, and patient-reported outcomes) and ensure minimal prerequisites for safe implementation (rehabilitation access, reliable follow-up/telecontact, proximity to a PCI-capable center, caregiver/transport, and a basic safety net with 48–72 h nurse calls and early clinic/tele-visits). Implementation will vary across health systems; barriers include access to cardiac rehabilitation, reliable telemedicine or follow-up channels, and geography (distance to PCI-capable centers). These contextual prerequisites should guide center-level adoption.


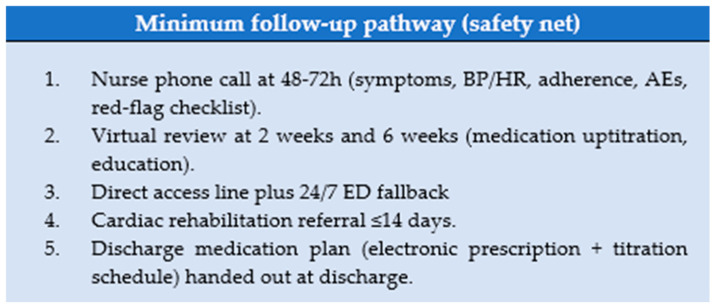


## 5. Conclusions

Early discharge is a safe and feasible management strategy with the appropriate profile of patients with ACS. Nonetheless, it requires good logistic organization in which coordination between hospital care, CRH units, and primary care centers should be fluid, bidirectional, and stable. The LATE2ACS protocol is proposed as a checklist that synthesizes the current evidence, being an easy, coherent, and intuitive tool that allows easy selection of the profile of patients requiring early discharge after ACS. On the other hand, this novel tool should be validated in a prospective evaluation to determine its true clinical and prognostic value, beyond logistical and functional considerations being taken into account. LATE2ACS is intentionally exploratory and should be interpreted as a pragmatic checklist summarizing current evidence rather than a validated score; prospective studies are needed before broad implementation. A pragmatic pilot (e.g., single-arm or stepped implementation with 30-day safety endpoints) would be an appropriate first step before a formal comparative study.

## Figures and Tables

**Table 1:** **TIMI risk scores for both UA/NSTEMI and STEMI.** The score for STEMI is a simple assessment based on clinical data at the time of patient arrival at the hospital.

**TIMI Risk Score**				**UA/NSTEMI** **0–2 points: low risk** **3–5 points: intermediate risk** **≥6 points: high risk**
**UA/NSTEMI**		**STEMI**		
	**POINTS**		**POINTS**	
Age ≥ 65 years	1	Age 65–74/>75	2/3	
≥3 CVRF	1	SBP < 100 mmHg	3	
Used ASA last 7 days	1	Heart rate > 100	2	
Prior CAD > 50% stenosis	1	Killip class ≥ 2	2	**STEMI** **0–4 points: low risk** **5–8 points: intermediate risk** **≥9 points: high risk**
>1 rest angina episode in <24 h	1	Anterior STEMI or LBBB	1	
ST-segment deviation	1	Diabetes, HTA, angina	1	
Elevated cardiac markers	1	Weight < 67 Kg	1	
**Total points**	**0–7**	Time to treat > 4 h	1	
		**Total points**	**0–14**	

**Table 2:** **Zwolle index.** Prognostic assessment of patients with acute myocardial infarction treated with primary angioplasty: implications for early discharge. Adapted from De Luca G., et al. [21].

Zwolle Risk Score		
	**Score**	
Killip class	1	+0
	2	+4
	3–4	+9
Final TIMI	0–1	+2
	2	+1
	3	+0
Age	≥60	+2
3v CAD	NO	+0
	YES	+1
Anterior AMI	NO	+0
	YES	+1
>4 h ischemia	NO	+0
	YES	+1
**Low risk: 0–3 points High risk: ≥ 4 points**		

**Table 3:** **Inclusion criteria for low-risk STEMI.** Predictors allowing early discharge after interventional treatment of acute coronary syndrome patients Adapted from Bauer D., et al. [29].

Low-Risk Profile Criteria in ACS
Age < 80 years KK-I at admission No CPR or NIMV Successful uncomplicated PCI with TIMI3 flow without significant residual stenosis (>90%) Absence of lesion in LMCA or 3v CAD No VT after 24 h from PCI LVEF ≥ 50% Hb > 11 g/dL at admission Glycemia < 270 mg/dL (≈15 mmol/L) eGFR > 60 mL/min/1.73 m^2^ Good social situation: close family support

**Table 4:** **CADILLAC risk score for ACS.** This score was developed to identify patients at low risk for adverse cardiovascular events following STEMI treated with PCI.

CADILLAC ACS Risk Score		
	**Points**	Patients are stratified into three risk groups that predict 30-day and 1-year mortality:
LVEF < 40%	4	
Renal failure	3	
Killip class 2 or 3	3	
Final TIMI < 3	2	**Low risk: 0–2 points** Up to 0.2% mortality at 30 days and up to 0–9% at 1 year **Intermediate risk: 3–5 points** Up to 1.9% mortality at 30 days and up to 4.5% at 1 year **High risk: ≥6 points** Up to 8.1% mortality at 30 days and up to 13.2% at 1 year
Age > 65 years	2	
Anemia	2	
3-vessel CAD	2	
Total points	0–14	

**Table 5:** **ACUITY-PCI risk score for NSTEMI.** This scoring system tool integrates clinical, angiographic, and laboratory/electrocardiographic variables specifically developed for patients with NSTEMI undergoing PCI [31].

ACUITY-PCI Risk Score (NSTEMI)		
	**Points**
Insulin-treated diabetes	12
Renal insufficiency	12
Troponin elevation or ST-segment deviation	8
Bifurcation lesion	4
Small-vessel/diffuse CAD	2
Extent of CAD (1 point for each 10 mm of disease)	1

**Table 6:** Criteria for low-risk profile in STEMI. Adapted from 2018 ESC/EACTS Guidelines on myocardial revascularization [26].

Low-Risk Criteria in STEMI (ESC)
LVEF > 40% by TTE or ventriculography. Successful primary angioplasty (TIMI 3). Absence of pending revascularization (surgical or percutaneous). Absence of persistent ischemia symptoms. Absence of HF (Killip–Kimball I). Absence of significant arrhythmias. Absence of hemodynamic instability. Absence of significant comorbidities. Circumstances of mobility and acceptable socio-family support.

**Table 7:** **Criteria for early discharge.** Safety of a Very Early Discharge Strategy for ST-segment Elevation Acute Coronary Syndrome. Adapted from: Marco Del Castillo Á. et al. [32].

Low-Risk Profile in STEMI
<65 years Complete or incomplete revascularization in case of distal disease or small caliber LVEF > 45% KK-I classification Admission to CRH program or early review (<3 weeks) Absence of CPA Absence of acute stent thrombosis

**Table 8:** **Comparison of risk scores and the novel LATE2ACS protocol.** This table shows the criteria that constitute the new LATE2ACS protocol and compares them with each of the criteria that define the previously validated risk scores. The green boxes represent those criteria that show consistency between the two scores, the light-green boxes show coincidences although with differences in the cut-off point or definition of the inclusion criterion, and the yellow ones refer to those parameters in which there is a very indirect coincidence in the criteria for inclusion. The blank spaces are those criteria in which there is no coincidence between the LATE2ACS protocol and each validated score.

				**GRACE 2007** [4]	**TIMI 2000** [2,30]	**ZWOLLE 2004** [21]	**CADILLAC 2005** [30]	**ACUITY 2012** [31]	**Marco Del Castillo 2019** [32]	**ESC Criteria 2019** [26]	**Bauer 2022** [29]
**L**	**Lungs**	Absence of heart failure (KK I)									
**A**	**Age and autonomy**	<80 years with favorable social situation									
**T**	**Final TIMI flow**	Restored final TIMI flow									
**E**	**Ejection fraction**	≥45%									
**2A**	**Absence of**	Anterior akinesia									
		Analysis abnormalities	Glycemia > 270 mg/dL								
			Hb < 11 g/dL								
			eGFR < 60 mL/min/1.73m^2^								
			Troponin peak reached								
**C**	**Coronary complication**	No complication related to PCI procedure									
**S**	**Shock and support**	NO shock, arrhythmia, arrest, support required									

**Table 9:** **LATE2ACS Decision Table (mandatory vs conditional thresholds).** Decision rule—all mandatory items must be met; conditional items resolved before discharge; organizational prerequisite secured. Key: M = mandatory; C = conditional; M-org = organizational mandatory.

Item	Threshold/Definition	Category	How to Verify	If NO
**Clinical stability**	Killip–Kimball I; no shock/NIMV/VT-VF	**M**	Clinical exam/monitoring	Not eligible
**Final TIMI flow**	TIMI 3 in culprit vessel	**M**	Cath report	Not eligible
**Procedural** **complications**	None (no-reflow sustained, perforation, acute ST, type 4a MI, major access)	**M**	Cath report	Not eligible
**Planned procedure**	No staged PCI/CABG	**M**	Team plan	Not eligible
**LVEF**	≥45%	**M**	TTE ≤24 h	Not eligible
**Renal function**	eGFR > 60 mL/min/1.73 m^2^	**M**	Same-day labs	Not eligible
**Anterior wall**	No anterior akinesia	**C**	TTE ≤24 h	Reassess 24 h
**Labs stability**	Hb ≥11 g/dL, glucose <270 mg/dL	**C**	Same-day labs	Correct/observe and re-check
**Troponin trend**	Peak reached (declining trend)	**C**	6–12 h post-PCI or 2× at 3–6 h	Repeat and defer
**Safety net**	48–72 h call; week-2 & week-6 reviews; CRH ≤ 14 d; 24/7 ED fallback	**M-org**	Appointments booked	Not eligible

**Table 10:** Anatomical and procedural exclusions for early discharge.

- LMCA culprit lesion at index event. - Incomplete revascularization with ischemia (residual stenosis ≥ 70% or abnormal FFR/iFR in a relevant vessel). - Periprocedural type 4a MI or sustained no-reflow, perforation, acute stent thrombosis, or major access-site complication. - Need for hemodynamic/respiratory support or persistent ventricular arrhythmias > 24 h post-PCI.

## Data Availability

No new data were generated or analyzed in this research.

## Abbreviations

The following abbreviations are used in this manuscript:

ACS	acute coronary syndrome
AMI	acute myocardial infarction
BP	blood pressure
CAD	coronary artery disease
CPA	cardiopulmonary arrest
CPR	cardiopulmonary resuscitation
CRH	cardiac rehabilitation
CVRF	cardiovascular risk factor
ED	emergency department
eGFR	estimated glomerular filtration rate
ESC	European Society of Cardiology
GRACE	Global Registry Acute Coronary Events
Hb	hemoglobin
HDI	hemodynamic instability
HF	heart failure
HR	heart rate
KDIGO	Kidney Disease: Improving Global Outcomes guidelines
KK	Killip–Kimball class
LBBB	left bundle branch block
LMCA	left main coronary artery
LV	left ventricular
LVEF	left ventricular ejection fraction
MACE	major adverse cardiac events
NIMV	non-invasive mechanical ventilation
NSTEMI	non-ST-elevation myocardial infarction
PCI	percutaneous coronary intervention
SBP	systolic blood pressure
STEMI	ST-elevation myocardial infarction
TIMI	thrombolysis in myocardial infarction, a grade of coronary flow
UA	unstable angina
